# Mapping of important taxonomic and productivity traits using genic and non-genic transposable element markers in peanut (*Arachis hypogaea* L.)

**DOI:** 10.1371/journal.pone.0186113

**Published:** 2017-10-17

**Authors:** Anil A. Hake, Kenta Shirasawa, Arati Yadawad, M. Sukruth, Malagouda Patil, Spurthi N. Nayak, S. Lingaraju, P. V. Patil, H. L. Nadaf, M. V. C. Gowda, R. S. Bhat

**Affiliations:** 1 Department of Biotechnology, University of Agricultural Sciences, Dharwad, India; 2 Department of Frontier Research, Kazusa DNA Research Institute, Chiba, Japan; 3 Department of Plant Pathology, University of Agricultural Sciences, Dharwad, India; 4 Department of Genetics and Plant Breeding, University of Agricultural Sciences, Dharwad, India; National Institute for Plant Genome Research, INDIA

## Abstract

A mapping population of recombinant inbred lines (RILs) derived from TMV 2 and its mutant, TMV 2-NLM was employed for mapping important taxonomic and productivity traits using genic and non-genic transposable element markers in peanut. Single nucleotide polymorphism and copy number variation using RAD-Sequencing data indicated very limited polymorphism between TMV 2 and TMV 2-NLM. But phenotypically they differed significantly for many taxonomic and productivity traits. Also, the RIL population showed significant variation for a few additional agronomic traits. A genetic linkage map of 1,205.66 cM was constructed using 91 genic and non-genic *Arachis hypogaea* transposable element (AhTE) markers. Using single marker analysis and QTL analysis, the markers with high phenotypic variance explained (PVE) were identified for branching pattern (32.3%), number of primary and secondary branches (19.9% and 28.4%, respectively), protein content (26.4%), days to 50% flowering (22.0%), content of oleic acid (15.1%), test weight (13.6%) and pod width (12.0%). Three genic markers (AhTE0357, AhTE0391, AhTE0025) with *Arachis hypogaea* miniature inverted-repeat transposable element (*AhMITE1*) activity in the genes *Araip*.*TG1BL* (B02 chromosome), *Aradu*.*7N61X* (A09 chromosome) and *Aradu*.*7065G* (A07 chromosome), respectively showed strong linkage with these taxonomic, productivity and quality traits. Since TMV 2 and TMV 2-NLM differed subtly at DNA level, the background noise in detecting the marker-trait associations was minimum; therefore, the markers identified in this study for the taxonomic and productivity traits may be significant and useful in peanut molecular breeding.

## Introduction

Peanut (*Arachis hypogaea* L.) is an important oilseed and legume crop apart from being a fodder crop. Peanut improvement through molecular breeding demands the development of genomic resources like quantitative trait loci (QTL) regions and their linked markers for various traits. Such a gene discovery approach can be best-employed with genotypes that differ for many traits but are genetically related [1]. Candidate regions or genes could be drastically reduced when a mutant was backcrossed to its non-mutagenized progenitor, and the segregants were used for mapping [2]. Such near isogenic lines have many advantages in fine-mapping of the target QTL by eliminating background noise [3].

In peanut, a narrow leaf mutant (NLM) was recovered from TMV 2, a Spanish bunch variety by EMS mutagenesis [4]. The mutant, TMV 2-NLM with linear lanceolate leaflets, belonged to Virginia runner type with semi-spreading growth habit and alternate branching pattern [5]. It showed several desirable characters such as high dry-matter production, high chlorophyll content, high dormancy, larger pod size and high test weight etc. [4]. TMV 2-NLM also showed high Rubisco content and less carbon discrimination [6]. Genetic analysis showed that, branching pattern, growth habit, leaflet shape were under the control of two genes each, while pod beak and pod constriction were under the control of three genes each with different gene action [5]. TMV 2-NLM recorded lower linoleic acid and higher oleic acid due to a point mutation in *AhFAD*2A gene as compared to TMV 2 [7].

Since TMV 2 and its mutant, TMV 2-NLM differed for several taxonomic and productivity traits, an attempt to compare their DNA sequence could reveal the regions contributing to the phenotypic changes. Also, the parent-mutant combination of TMV 2 and TMV 2-NLM make ideal parents for developing a mapping population. With these advantages, a recombinant inbred line population was developed at University of Agricultural Sciences Dharwad, India by crossing TMV 2 with TMV 2-NLM [5].

Mapping population is generally genotyped with varieties of markers [8]. *Arachis hypogaea* transposable element (AhTE) marker system, which scans both genic and non-genic regions for the polymorphism due to the insertion of *Arachis hypogaea* miniature inverted-repeat transposable element (*AhMITE1*) at a higher rate [9], is expected to identify the markers linked/associated with the important traits. Further, availability of the gene prediction data from *Arachis duranensis* (A genome donor) and *Arachis ipaensis* (B genome donor), the two progenitors of cultivated peanut would enable the identification of candidate protein coding gene(s) and RNA coding genes that are involved in differential transpositions leading to phenotypic variations. Therefore, an effort was made in this study to look for the phenotypic and genotypic polymorphism between TMV 2 and TMV 2-NLM, and to map important traits using the RIL population of TMV 2 × TMV 2-NLM. A few QTL regions and genes contributing for the traits were identified.

## Materials and methods

### Comparison of TMV 2 and TMV 2-NLM

TMV 2 and TMV 2-NLM were evaluated in the field over two seasons (rainy seasons of 2014 and 2015) for various taxonomic and productivity traits. They were subjected for DNA sequence comparison using the ddRAD-Seq method of reduced genome representation sequencing to detect single nucleotide polymorphisms (SNPs) and copy number variations (CNVs) as explained earlier [10, 11]. The data for ddRAD-Seq was registered in a public DNA database, DDBJ Sequence Read Archive (http://www.ddbj.nig.ac.jp), under the accession number of DRA005804. These SNPs were checked for their position and functional annotation using the gene prediction data from the diploid genomes (available at https://peanutbase.org) as described earlier [11]. TMV 2 and TMV 2-NLM were also checked for the differential activity of the *AhMITE1* at various sites [9, 12] in the genome. The polymorphic sites were searched for functional annotation.

### Evaluation of the RIL mapping population

The mapping population with 432 RILs derived from TMV 2 and its EMS-derived mutant, TMV 2-NLM [4] was developed by single seed descent method at University of Agricultural Sciences (UAS), Dharwad, India [5]. The F_14_ seeds of these RILs were obtained from the Department of Genetics and Plant Breeding, UAS, Dharwad, India.

#### Phenotyping of the RILs

The RIL population along with the parents was grown at IABT Garden of the Department of Biotechnology, UAS, Dharwad, India during the rainy seasons of 2014 and 2015 in a randomized block design with two replications. Each replication consisted of 2 rows of 1.5 mt length with a spacing of 30 cm × 10 cm. Five representative plants were selected randomly from each RIL to record the taxonomic and productivity traits. Reactions to late leaf spot (LLS) and rust were assessed by subjecting the RILs to field screening following spreader row technique [13] in which the disease spreader plants [TMV 2 and Mutant 28–2 [14]] were planted at regular interval of 10 rows, and the disease epiphytotic condition was created using the inoculums. Disease scoring for both LLS and rust was done at 70, 80 and 90 days after sowing (DAS) according to modified 9-point scale [15]. Observations on taxonomic and morphological traits (branching pattern, growth habit, plant height, leaflet length, leaflet width, leaflet shape, leaflet colour), and productivity traits (number of pods per plant, pod yield per plant, pod yield, test weight, shelling percentage, pod length, pod width, pod size, pod constriction, pod reticulation, kernel colour, seed shape, seeds per pod) were recorded as per the groundnut descriptor [16]. Number of primary branches (NPB) borne on main axis, and the number of secondary branches (NSB) borne on primary branches were recorded. Sound mature kernel weight (%) was calculated as weight of well-developed kernels from a unit weight of kernels. Nutritional parameters contents of protein, oil, arachidic acid, behenic acid, eicosanoic acid, lignoseric acid, linoleic acid, oleic acid and palmitic acid were analyzed by near infrared spectroscopy (NIRS) using FOSS NIR System, 6500 Composite (FOSS Analytical A/S, Denmark). Chlorophyll content was measured in terms of SPAD chlorophyll meter reading (SCMR) with the help of SPAD meter on 37 DAS. Seed dormancy test was conducted by subjecting the dried seeds for germination after 15 days of harvesting, and observing for the number of seeds germinated on each day for 14 consecutive days. Percentages of seeds germinated were used to record the level of seed dormancy using the standard scores [17].

### Genotyping of the RILs

Total genomic DNA from the RILs was extracted from young leaves using modified cetyl trimethyl ammonium bromide (CTAB) method [18]. The RILs were genotyped with the AhTE markers, which were polymorphic between TMV 2 and TMV 2-NLM (S1 Table). The PCR was carried out in a reaction volume of 10 μl with 50 ng of template DNA, 5 pmol of each primer, 10X of *Taq* polymerase buffer [500 mM KCl, 100 mM Tris-HCI (pH 8.5], 2.0 mM of MgCl_2_, 0.25 mM of dNTPs and 0.15 U of *Taq* polymerase. PCR was performed in 96-wellplates using Veriti 96-Well Thermal Cycler (Applied Biosystem) with the temperature profile of 95°C for 5 min and 35 cycles of 95°C for 1 min, 53°C for 1 min and 72°C for 1.30 min, and 72°C for 8 min for final extension. The PCR products were analyzed by loading them on 1.8% agarose gel and electrophoresing in 1X TAE at 80 V for 2 h using Bio-Rad gel electrophoresis unit. The amplicons were visualized using ethidium bromide staining method. Specific PCR product was identified for each marker [9, 12] and the alleles differing for 205 bp (equal to the size of *AhMITE1*) were scored. RILs were scored as A [homozygote as the first parent (TMV 2)], B [homozygote as the second parent (TMV 2-NLM)], H (heterozygote), C [not genotype a (b-allele is dominant)] and D [not genotype b (a-allele is dominant)] as per JoinMap 4 format [19],

### Single marker analysis (SMA)

Single marker analysis was performed to find out the association between the AhTE markers and the traits observed in this study by calculating *F* statistic and simple linear regression coefficient [20] using WinQTL Cartographer version 2.5 [21]. Those significant and major markers showing >10% R^2^ were analyzed for their position in the genome and functional annotation using the gene prediction data from the diploid genomes (available at https://peanutbase.org).

### Linkage map construction

Linkage analysis was performed with JoinMap 4.0 [19]. The “Locus genotype frequency” function was applied to calculate chi-square values for each marker to test for the expected 1:1 segregation. Markers were placed onto linkage groups with the “LOD groupings” and “Create groups for mapping” command using the Kosambi map function [22]. Calculation parameters were set for a minimum LOD threshold of 3.0, and the marker order in groups was established by “Calculate Map” command. After developing the framework genetic map, the unmapped markers were placed onto different linkage groups. The linkage map was drawn using the software MapChart 2.2 [23].

### QTL analysis

The QTL mapping was performed for the phenotypic data collected during the two seasons; the rainy seasons of 2014 and 2015 and the linkage map using Windows QTL Cartographer version 2.5 [21] to detect QTL regions. Composite interval mapping (CIM) with 1,000 permutations, 1.0 cM scanning interval between markers and putative QTL, and a window size of 10.0 cM was used for QTL mapping.

## Results

**Fig 1:**
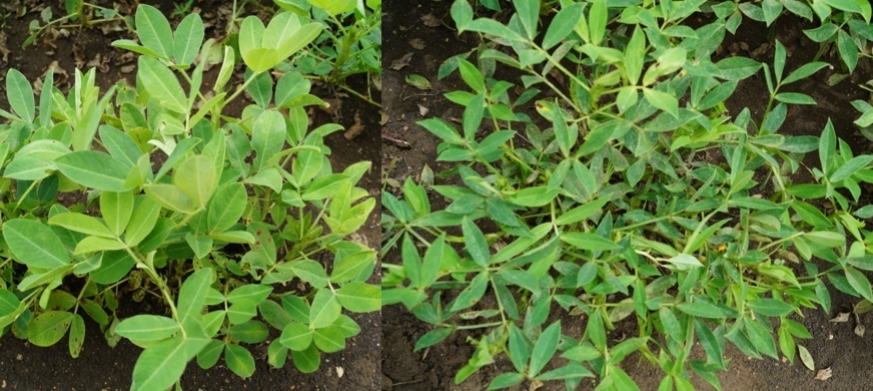
TMV 2 (left) and its mutant TMV 2-NLM (right).

Field evaluation of TMV 2 and its mutant, TMV 2-NLM during the rainy seasons of 2014 and 2015 showed main stem flowering, sequential branching pattern and erect growth habit and for TMV 2, and absence of main stem flowering, alternate branching pattern and semi-spreading growth habit for TMV 2-NLM (Table 1 and Fig 1). TMV 2 had wide elliptical leaflets, while TMV 2-NLM had narrow, linear and lanceolate leaflets. Significant differences were also observed between TMV 2 and TMV 2-NLM for number of primary branches, number of secondary branches, pod yield per plant, test weight, shelling percentage, sound mature kernel weight, arachidic acid, behenic acid, eicosenoic acid, lignoseric acid, linoleic acid, oleic acid, palmitic acid and seed dormancy. Thus, TMV 2 and its primary mutant TMV 2-NLM differed for several taxonomic, agronomic, productivity and nutritional traits.

**Table 1:** Distinguishing features of TMV 2 and TMV 2- NLM.

SN	Name of the trait	TMV 2	TMV 2- NLM
	**Taxonomic traits**		
**1**	Branching pattern	Sequential	Alternate
	**Morphological traits**		
**2**	Growth habit	Erect	Decumbent-3 (Semi-spreading)
**3**	Days to 50% flowering	31	36
**4**	Leaflet shape	Wide-elliptic	Linear lanceolate
**5**	Leaflet colour	Light green	Dark green
**6**	Leaflet length (cm)	5.57	4.76
**7**	Leaflet width (cm)	2.48	1.25
**8**	Number of primary branches	5.73	9.00
**9**	Plant height (cm)	35.58	28.69
**10**	Number of secondary branches	0.00	9.25
	**Physiological traits**		
**11**	Seed dormancy Score (0 to 8)	Absent (6)	Present (3)
**12**	SPAD Chlorophyll Meter Reading (SCMR)	24.59	44.56
	**Productivity traits**		
**13**	Pod size	Medium	Large
**14**	Number of pods per plant	24.58	11.9
**15**	Pod yield per plant (g)	16.55	7.83
**16**	Pod yield (Kg/ha)	4883	2043
**17**	Test weight	37.00	45.93
**18**	Shelling percentage	74.60	66.53
**19**	Kernel colour	Light tan	Dark tan
**20**	Pod beak	slight	Moderate
	**Nutritional traits**		
**21**	Protein (%)	32.27	27.50
**22**	Oil (%)	46.58	43.94
**23**	Linoleic acid (%)	35.82	27.76
**24**	Oleic acid (%)	42.38	54.47
**25**	Arachidic acid (%)	1.99	1.55
**26**	Behenic acid (%)	3.52	2.75
**27**	Palmitic acid (%)	12.37	11.50
**28**	Stearic acid (%)	4.00	3.15

**Fig 2:**
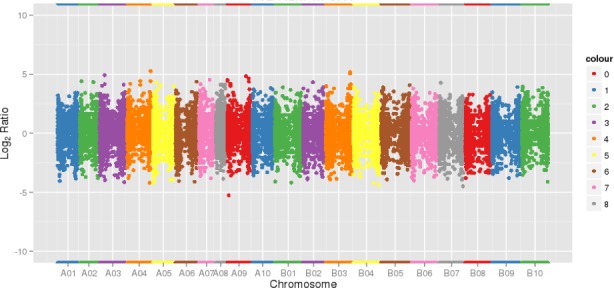
Copy number variation in TMV 2-NLM when compared to TMV 2.

An attempt was made to check the genetic differences between TMV 2 and TMV 2-NLM. ddRAD-Sequencing of TMV 2 and TMV 2-NLM with ~4X coverage could detect a total of 31 SNPs across 7 chromosomes (Table 2). Of them, only three SNPs were found from A genome, while remaining 28 SNPs originated from B genome. Twenty-nine SNPs were genic and only two were non-genic with respect to their location. Of the genic SNPs, a large number (17) was present in the introns. Of the six SNPs present in the exonic region, four were non-synonymous and two were synonymous. It was interesting to note that a few genes accumulated more SNPs upon EMS mutagenesis. *Araip*.*NZ9YG* gene on chromosome B04 coding for a protein with “F-box protein interaction domain” carried 14 SNPs, while *Araip*.*X5KQ1* gene on chromosome B01 coding for probable sugar phosphate-protein carried four SNPs. In total, nine genes showed sequence alterations due to SNPs with or without possible functional alterations. An effort was made to check the copy number variations (CNVs) between TMV 2 and TMV 2-NLM. A total of 1,200 genomic regions showed significant CNVs (Fig 2), however, only five regions showed a change (increase or decrease) by at least five-fold of log_2_.

**Table 2:** Identification of SNPs between TMV 2 and TMV 2-NLM.

SN	Gene	Chromosome	No. of SNPs	SNP location	Annotation	Transcript
**1**	Aradu.EGH8I	A01	01	Intron	Adenine phosphoribosyl transferase-like protein	EZ725386.1 [24]
**2**	Aradu.KAB8X	A07	01	3'UTR (954 bp apart)	Disease resistance protein (CC-NBS-LRR class) family protein; IPR002182 (NB-ARC), IPR027417 (P-loop containing nucleoside triphosphate hydrolase); GO:0043531 (ADP binding)	EZ742624.1 [24]
**3**	Aradu.MA5IZ	A06	01	Exon (synonymous)	GYF domain-containing protein; IPR003169 (GYF); GO:0005515 (protein binding)	Adur5580_comp0_c0_seq5 [25]
**4**	Araip.G5J45	B10	01	Exon (non-synonymous)	Transmembrane protein, putative	Aipa26504_comp0_c0_seq1 [25]
**5**	Araip.K4U0Q	B10	05	Downstream (2546 to 2692 bp apart)	Pentatricopeptide repeat (PPR) superfamily protein; IPR002885 (Pentatricopeptide repeat), IPR011990 (Tetratricopeptide-like helical), IPR027434 (Homing endonuclease); GO:0004519 (endonuclease activity), GO:0005515 (protein binding)	gi|372391829|gb|JR544303.1| [26]
**6**	Araip.NZ9YG	B04	14	Intron	F-box protein interaction domain protein; IPR001810 (F-box domain), IPR011043 (Galactose oxidase/kelch, beta-propeller), IPR017451 (F-box associated interaction domain); GO:0005515 (protein binding)	Aipa36174_comp0_c1_seq4 [25]
**7**	Araip.X5KQ1	B01	05	Exon [synonymous (1) and non-synonymous (2)] and intron (2)	Probable sugar phosphate/phosphate translocator [Glycine max]; IPR004853 (Triose-phosphate transporter domain)	GG14274|comp0_c0_seq1 [25]
**8**	Araip.ZEW2Y	B10	1	Exon (non-synonymous)	Cyclophilin-like peptidyl-prolyl cis-trans isomerase family protein; IPR002130 (Cyclophilin-type peptidyl-prolyl cis-trans isomerase domain), IPR011990 (Tetratricopeptide-like helical); GO:0003755 (peptidyl-prolyl cis-trans isomerase activity), GO:0005515 (protein binding), GO:0006457 (protein folding)	EZ734646.1 [24]

TMV 2 and TMV 2-NLM were also checked for the differential activity of *AhMITE1* over 369 genomic sites using the AhTE markers. Polymorphism was observed at 105 (28.4%) sites between TMV 2 and TMV 2-NLM. Of them, 57 sites represented genic regions, while 48 belonged to non-genic regions. Sequence analysis indicated that out of the 57 genic sites, the transpositional activity of the *AhMITE1* was found in the exons at six sites. In 16 genic sites, the transposon activity was restricted to intronic regions. Six genes had transposition site at untranslated regions (UTRs). Fifteen genes had transposition site at upstream regions (23–949 bp) and 14 genes had transposon activity at downstream regions (8–934 bp). Overall, the phenotypic and genotypic data revealed very limited genotypic polymorphism between TMV 2 and TMV 2-NLM, despite significant phenotypic differences.

In order to map the genomic regions governing taxonomic and productivity traits, the mapping population of TMV 2 × TMV 2-NLM was employed. One hundred and five AhTE markers showing polymorphism between TMV 2 and TMV 2-NLM were used for genotyping the 432 RILs. A linkage map of 1,205.66 cM was constructed with 91 markers on 20 linkage groups (LGs) (S2 and S3 Tables). The length of LGs ranged from 4.59 cM (A04) to 164.12 cM (B09). The number of markers mapped on the linkage groups ranged from 2 (A02a, A04, A05a, A07 and B10) to 11 (A09 and B09). The overall inter-marker distance was 18.13 cM.

RILs were field-evaluated during the rainy seasons of 2014 and 2015. They differed significantly for most of the taxonomic, agronomic, productivity and nutritional traits (S4 Table). High PCV and GCV were observed for LLS score at 80 and 90 DAS, number of pods per plant, pod yield per plant, number of secondary branches and seed dormancy, for which the parents also differed significantly (S5 Table). However, the RILs also showed considerable variability for resistance to late leaf spot and rust, pod and seed features (pod constriction, pod reticulation and seed shape) and some fatty acids (behenic and eicosenoic acid), though the parents did not differ significantly.

**Fig 3:**
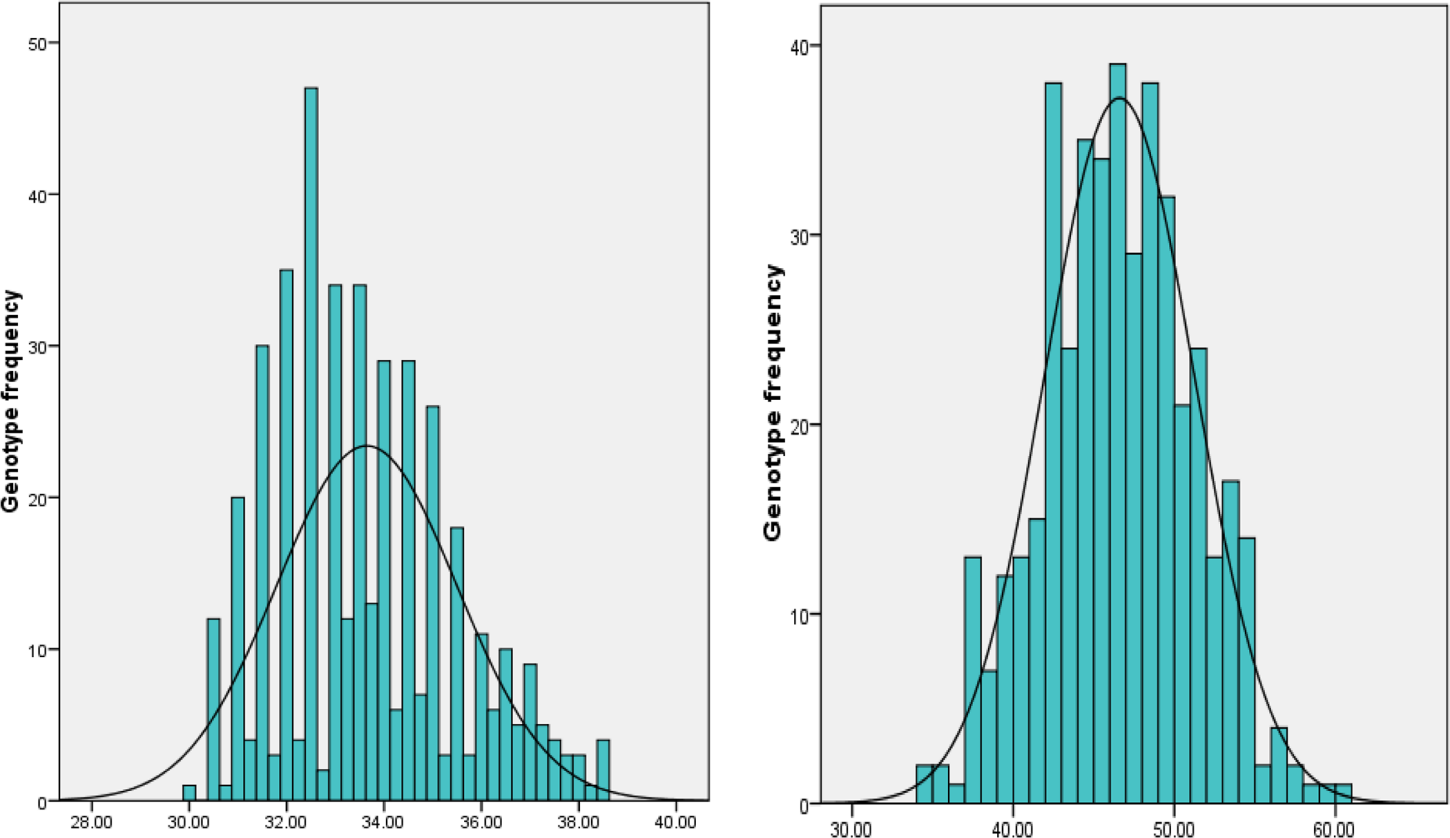
Frequency distribution of RILs of TMV 2 × TMV 2-NLM for days to 50% flowering (a) and oleic acid (b).

Majority of the traits showed normal distribution as tested by skewness and kurtosis (S6 Table and Fig 3). Correlation analysis indicated a positive and significant association of days to 50% flowering with the number of primary and secondary branches (S7 Table). But days to 50% flowering recorded negative and significant correlation with productivity and oil quality traits. Test weight showed positive and significant association with pod yield and content of oleic acid palmitic acid and O/L ratio. Test weight was also positively and significantly correlated with pod width. It was also observed that the sequential type of branching habit resulted in higher pod yield.

Single marker analysis (SMA) was performed to find any association between the AhTE markers and the traits. A total of 41 and 43 traits in 2014 and 2015, respectively were subjected for SMA (S8 and S9 Tables). Three markers (AhTE0357, AhTE0391 and AhTE0523) showed significant association and high R^2^ with one or more traits during 2014 and/or 2015 (Table 3). AhTE0357 showed the highest R^2^ (32.8%) for the branching pattern (sequential/alternate). AhTE0357 also showed strong association with days to 50% flowering and number of primary and secondary branches. AhTE0391 showed strong association with the contents of protein, oil, oleic acid, linoleic acid and palmitic acid. Apart from these three markers, AhTE0025 showed high R^2^ with test weight and pod width.

**Table 3:** Single marker analysis for important agronomic, productivity, nutritional and disease resistance traits in peanut.

SN	Marker	Trait	Rainy season of 2014			Rainy season of 2015		
			F	p	PVE (%)	F	p	PVE (%)
**1**	**AhTE0357**	BP	198.7	0.0	32.3	198.7	0.0	32.3
		LS	52.2	0.0	10.0	52.2	0.0	10.0
		50%F	70.3	0.0	14.2	64.2	0.0	12.4
		NPB	12.9	0.0	2.8	112.8	0.0	20.0
		NSB	53.2	0.0	10.8	125.8	0.0	23.3
**2**	**AhTE0391**	OIL	36.1	0.0	7.7	26.5	0.0	5.7
		OLE	61.3	0.0	12.4	65.0	0.0	13.1
		LIN	67.8	0.0	13.5	66.9	0.0	13.4
		O/L	59.3	0.0	12.1	59.8	0.0	12.1
		PAL	48.7	0.0	10.2	45.2	0.0	9.5
**3**	**AhTE0523**	ECO	72.4	0.0	14.4	68.9	0.0	13.8
		STE	56.2	0.0	11.6	6.6	0.0	1.5
		PROT	49.1	0.0	10.2	16.7	0.0	3.7
		PW	11.2	0.0	2.5	55.2	0.0	11.4
		LIG	39.2	0.0	8.4	65.8	0.0	13.3
		LIN	50.4	0.0	10.5	43.6	0.0	9.2
**4**	**AhTE0025**	TW	31.8	0.0	6.9	36.6	0.0	7.8
		PW	45.6	0.0	9.6	39.6	0.0	8.4

BP: Branching pattern, LS: Leaflet shape, 50%F: Days to 50% flowering, NPB: Number of primary branches, NSB: Number of secondary branches, OIL: Oil content, OLE: Oleic acid, LIN: Linoleic acid, O/L: Oleic to linoleic acid ratio PAL: Palmitic acid, ECO: Eicosenoic acid, STE: Stearc acid, PROT: Protein, LIG: Lignoceric acid, TW: Test weight, PW: Pod width and PVE: Phenotypic variance explained

**Fig 4:**
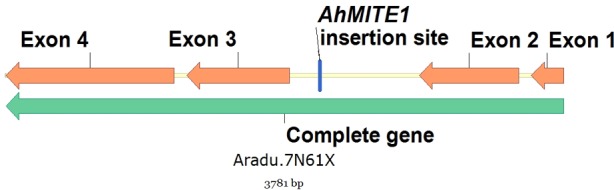
Insertion of *AhMITE1* in the intronic region of the gene *Aradu*.*7N61X*.

Analysis of the genomic position of these four markers showed that AhTE0357, AhTE0391 and AhTE0025 were genic. The transposition site of *AhMITE1* at AhTE0357 locus was located at 79 bp downstream of the gene *Araip*.*TG1BL*. The marker locus corresponding to AhTE0391 coincided with the gene *Aradu*.*7N61X*. The *AhMITE1* transposition site was present at 2,129 bp of the second intronic region of *Aradu*.*7N61X* (Fig 4). At AhTE0025 locus, *AhMITE1* was inserted in the intronic region of *Aradu*.*7065G*. But, the marker AhTE0523 showed several significant hits upon BLAST, therefore the exact position could not be decided. Function prediction revealed that *Aradu*.*7N61X* codes for alpha-glucosidase, while *Aradu*.*7065G* and *Araip*.*TG1BL* code for aldo/keto reductase family oxidoreductase and unknown protein (galactose oxidase/kelch, beta-propeller), respectively.

**Fig 5:**
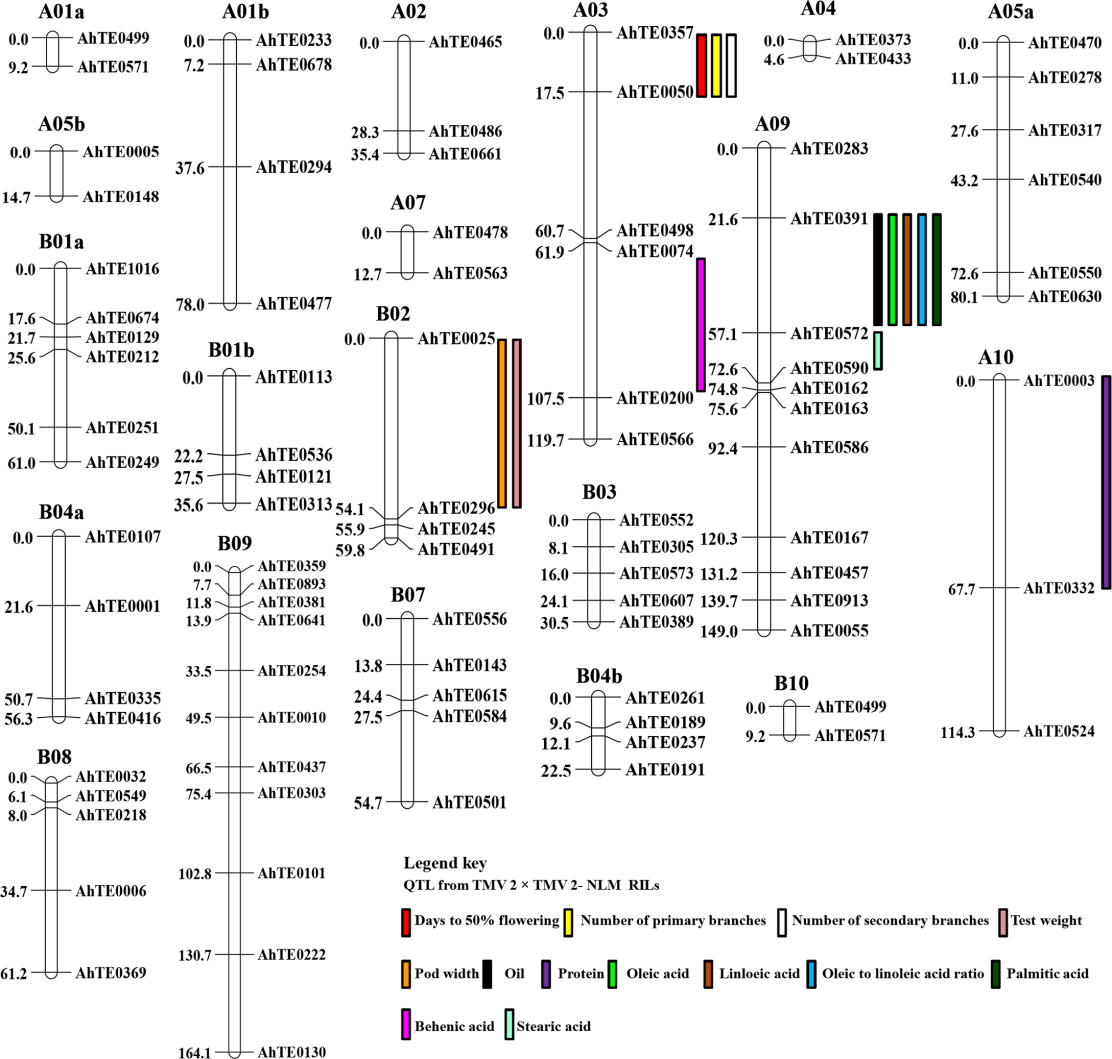
QTL map for agronomic, productivity and nutritional traits from the RILs of TMV 2 × TMV 2-NLM.

QTL analysis was attempted using the linkage map and the phenotypic data for 31 traits in 2014, and 33 traits in 2015 (S10 and S11 Tables). The QTL map showed seven major (PVE more than 10%) QTL regions for 22 traits across the years (Table 4 and Fig 5). The number of traits governed by these QTL ranged from one (AhTE0074-AhTE0200 and AhTE0005-AhTE0148) to nine (AhTE0391-AhTE0572).

**Table 4:** Major effect QTLs identified for important agronomic, productivity and nutritional traits in peanut.

SN	QTL in the marker interval	Linkage group	Marker interval (cM)	Trait	Rainy season of 2014			Rainy season of 2015		
					LOD	Ae	PVE (%)	LOD	Ae	PVE (%)
**1**	**AhTE0357-AhTE0050**	A03	0.00–17.52	50%F	13.8	-0.8	18.5	14.3	-1.0	22.0
		A03	0.00–17.52	NPB	11.6	-0.1	1.0	22.8	-1.7	19.9
		A03	0.00–17.52	NSB	18.5	-2.9	10.8	26.2	-1.0	28.4
**2**	**AhTE0003-AhTE0332**	A10	0.00–67.66	PROT	9.5	1.4	25.4	11.2	1.8	26.4
**3**	**AhTE0391-AhTE0572**	A09	21.57–57.11	OIL	9.1	0.5	10.4	6.1	0.5	6.7
		A09	21.57–57.11	OLE	10.4	-1.2	12.4	11.6	-1.4	15.1
		A09	21.57–57.11	LIN	11.4	0.6	15.5	11.1	0.6	14.8
		A09	21.57–57.11	O/L	10.8	0.0	15.0	11.0	0.0	15.2
		A09	21.57–57.11	PAL	9.8	0.2	14.8	10.7	0.2	15.3
**4**	**AhTE0572-AhTE0590**	A09	57.11–72.62	STE	7.4	0.1	12.2	2.9	0.1	4.6
		A09	57.11–72.62	PW	-	-	-	7.8	-0.05	10.9
**5**	**AhTE0025-AhTE0296**	B02	0.00–54.14	TW	8.2	-2.3	8.9	10.6	-4.0	13.6
		B02	0.00–54.14	PW	9.8	0.0	9.3	12.0	-0.1	11.6
**6**	**AhTE0074-AhTE0200**	A03	61.94–107.48	BEH	4.5	0.1	7.1	4.5	0.1	11.8
**7**	**AhTE0005-AhTE0148**	A05	0.00–14.69	PW	-	-	-	13.32	-0.07	15.9

50%F: Days to 50% flowering, NPB: Number of primary branches, NSB: Number of secondary branches, PROT: Protein content, OIL: Oil content, OLE: Oleic acid, LIN: Linoleic acid, O/L: Oleic to linoleic acid ratio, PAL: Palmitic acid, STE: Stearic acid, PW: Pod width, TW: Test weight, BEH: Behenic acid, cM: centiMorgan, LOD: logarithm of the odds, Ae: Additive effect and PVE: Phenotypic variance explained

QTL flanked by AhTE0357-AhTE0050 showed high PVE for days to 50% flowering, number of primary and secondary branches (Table 4). QTL between AhTE0003-AhTE0332 showed PVE of 26.0% and 26.4% for protein content during 2014 and 2015, respectively. QTL flanked by AhTE0025-AhTE0296 showed a PVE of 13.6% and 11.6% for test weight and pod width, respectively during 2015. Similarly, QTL at AhTE0391-AhTE0572 governed oil content, linoleic acid, oleic acid palmitic acid and O/L ratio. An effort was made to find out the gene content in the QTL region AhTE0391-AhTE0572. A total of 256 genes were predicted in the region homeologous to AhTE0391-AhTE0572 on B09 chromosome. Identifying and dissecting these genes for other QTL regions to find out the candidate gene(s) requires additional experimental evidence.

TMV 2 contributed the favourable allele at AhTE0003-AhTE0332 for protein content. The allele contributed by TMV 2 at AhTE0357-AhTE0050 resulted in reduced days to 50% flowering and number of primary branches. TMV 2-NLM contributed favourable allele at AhTE0391-AhTE0572 for oleic acid content. For test weight, TMV 2-NLM contributed the favourable allele at AhTE0025-AhTE0296.

An attempt was made to select the superior RILs for variety development and commercial release. High heritability and genetic advance over mean (GAM) were observed for LLS score at 90 DAS, days to 50% flowering, test weight, pod width, oil and protein content, oleic acid, linoleic acid and SCMR. Fifteen RILs were marginally superior for pod yield/plant over the best parent, TMV 2 (Table 5). However, only one RIL [2-19(f)] was significantly superior over TMV 2 for pod yield. The RIL 2-45(a) was superior over TMV 2 for pod yield, days to 50% flowering (early), test weight, sound mature kernel weight, protein content, oil content, oleic acid, O/L ratio and LLS and rust resistance. Another RIL 1-11(b) was superior over TMV 2 for pod yield, seed dormancy (moderate), days to 50% flowering (early), test weight, sound mature kernel weight, oleic acid, and O/L ratio. RILs 2-14(a)(ii), 2-29(g), 2-33(a) and 2-23(c) were superior over TMV 2 for pod yield, oil content, oleic acid, test weight, sound mature kernel weight and days to 50% flowering (early). Of these 15 RILs, four foliar disease resistant lines [2–45 (a), 2–25 (a), 2-14(a)(i) and 2-76(b)] were subjected for RAD-Seq, and compared with TMV 2 at nearly 28 Mb random nucleotide positions. RIL 2–45 (a) showed the highest similarity (4 SNP) with TMV 2 followed by 2–25 (a) (5 SNP), 2-14(a)(i) (8 SNP) and 2-76(b) (30 SNP). At *Aradu*.*A01_2581365*, *Aradu*.*A06_112199516*, *Araip*.*B02_34272578* and *Araip*.*B10_2552983* SNP sites, the four-foliar disease resistant lines shared the same nucleotide in contrast to TMV 2, indicating a possible co-segregation between SNP and resistance to LLS and rust.

**Table 5:** Performance of superior RILs selected from TMV 2 × TMV 2-NLM mapping population.

SN	RIL	PY	NPPP	SP	TW	SMKW	LLS 90	Rust 90	50% F	DORM	OIL	PROT	OLE	O/L	PC	PR	PB	PS
1	2-19(f)	6508.52	22.39	69.42	48.02	98.23	7.50	7.00	33.25	3.00	44.99	29.14	54.64	1.99	Moderate	Prominent	Slight	Medium
2	1-31(f)	5849.89	14.00	72.18	46.38	97.42	7.00	5.50	33.50	3.50	45.20	31.80	45.75	1.36	Moderate	Slight	Slight	Medium
3	2-14(a)(i)	5471.71	13.58	71.84	49.53	97.47	7.25	4.50	31.00	1.00	43.35	28.59	46.49	1.41	Slight	Moderate	Absent	Large
4	2-24(c)	5561.45	18.80	70.63	55.44	97.93	3.50	4.00	35.00	0.00	46.09	31.10	57.14	2.44	Moderate	Moderate	Moderate	Large
5	2-14(a)(ii)	5597.27	20.28	70.02	42.73	99.90	7.00	5.25	31.00	1.50	46.08	28.25	47.80	1.49	Moderate	Moderate	Slight	Large
6	2-33(a)	5387.88	22.68	72.56	35.38	98.97	5.00	5.50	31.50	0.00	48.45	29.40	47.58	1.45	Moderate	Slight	Slight	Medium
7	2-13(a)	5851.11	17.88	66.09	39.82	98.78	7.00	6.50	33.00	3.50	47.62	33.56	40.65	1.10	Moderate	Slight	Slight	Medium
8	2-29(g)	5234.94	22.08	73.14	50.08	98.14	6.75	6.75	31.00	3.00	47.65	30.44	48.52	1.57	Moderate	Slight	Slight	Large
9	2-76(b)	5364.43	15.65	70.99	56.54	98.92	4.75	3.50	35.25	0.00	44.12	28.08	46.25	1.37	Slight	Slight	Absent	Large
10	2-45(a)	5209.09	18.80	73.09	58.25	99.06	1.75	4.75	31.00	0.00	46.67	32.79	47.90	1.58	Moderate	Slight	Slight	Large
11	1-11(b)	5027.00	10.17	75.71	52.89	99.11	7.00	5.75	31.25	4.00	44.96	31.94	49.08	1.63	Moderate	Moderate	Slight	Medium
12	2-60(f)	5573.46	22.30	68.11	47.34	95.04	6.75	6.75	34.00	0.00	45.93	30.63	50.26	1.67	Moderate	Slight	Slight	Large
13	2-5(c)	5033.05	18.98	73.45	31.30	96.31	7.50	6.75	32.50	0.00	49.60	31.36	53.39	1.83	Moderate	Slight	Slight	Medium
14	2-72(f)	4925.06	19.20	75.01	41.01	97.72	8.00	7.00	32.00	0.00	47.45	34.83	37.84	1.02	Moderate	Slight	Slight	Medium
15	2-23(c)	5309.72	20.13	68.89	45.37	98.67	7.25	7.00	35.00	3.00	46.60	32.05	51.26	1.75	Moderate	Slight	Slight	Medium
	TMV2	4883.52	24.59	74.12	36.25	95.84	7.13	6.25	32.50	2.75	46.58	32.13	42.48	1.20	Moderate	Slight	Slight	Medium

PY: Pod yield, NPPP: Number of pods per plant, SP: shelling percentage, TW: Test weight, SMKW: Sound mature kernel weight, LLS 90: Late leaf spot at 90 DAS, Rust 90: Rust at 90 DAS, 50%F: Days to 50% flowering, DORM: Seed dormancy, OIL: Oil content, PROT: Protein content, OLE: Oleic acid, O/L: Oleic to linoleic acid ratio, PC: Pod constriction, PR: Pod reticulation, PB: Pod beak and PS: Pod size

## Discussion

TMV 2, a popular and elite variety of peanut, and its EMS-derived mutant TMV 2-NLM were used to identify the genomic regions contributing to the important taxonomic and productivity traits in peanut by QTL mapping using a large number (432) of their recombinant inbred lines (RILs). TMV 2 and TMV 2-NLM differed significantly for the growth habit, number of branches (primary and secondary), productivity traits and quantity and quality of the oil. The RIL population showed considerably high variability not only for those traits for which the parents differed, but also for a few other traits (resistance to LLS and rust, pod and kernel features), thus allowing QTL detection for several traits. RAD-Sequencing, which is a reduced representation of genome sequencing method evaluating ~1% of the genome, revealed genetic differences in terms of SNPs and copy number variations. The density of SNP was about 1 per Mb, which was comparable to the density found in tomato mutants [11]. Of the total 31 SNPs, only four were non-synonymous, indicating very few functional differences between TMV 2 and TMV 2-NLM. Likewise, significant copy number variations were only five within 1% of the genome. Though SNPs and CNVs can have significant effect on phenotypes through expression variation [27–29], TMV 2 and TMV 2-NLM did not differ significantly since they showed very few differences at DNA sequence. TMV 2 and TMV 2-NLM also showed the differential transpositional activity of *AhMITE1* at 105 loci out of 369 loci tested in this study. Further, it was found that *AhMITE1* activity was restricted to exons of only six genes. Thus, TMV 2 and TMV 2-NLM could constitute ideal parents of a RIL population which can be employed for mapping the traits by reducing the background noise.

Using 91 AhTE markers, a partial linkage map of 1,205.66 cM was constructed in a new RIL mapping population (TMV 2 × TMV 2-NLM) in peanut. The marker order was grossly comparable to the maps for SKF2 (Satonoka × Kintoki) and NYF2 (YI-0311 × Nakateyutaka), which were also constructed with TE markers [12]. The markers mapped on A01, A02, A04, A05, B01, B03, B04 and B07 could be confirmed with BLAST. But other markers could not be confirmed since they differed partly (differing for homeologous chromosomes) or completely. Further, a QTL map was constructed using this linkage map along with the phenotypic data on taxonomic and productivity traits over two seasons (rainy seasons of 2014 and 2015). Major QTL regions were identified for days to 50% flowering, number of branches, test weight, pod width, and contents of protein, oil, oleate, linoleate, O/L ratio and palmitate. A QTL region with AhTE0391 and AhTE0572 as the flanking markers (on A09) had PVE of 13.40%, 15.42% and 20.53% towards oleic acid (O), linoleic acid (L) and O/L ratio, respectively. AhTE0391 and AhTE0572 had a distance of 0.50 Mb and 5.6 Mb, respectively from the *AhFAD2A* on chromosome A09. *AhFAD2A* and *AhFAD2B* (on B09) are the main genes [30], though many genes contribute to oleic acid and linoleic acid content and O/L in peanut [31].

The markers flanking the QTL were also found to be strongly associated with the same traits when checked with single marker analysis. The marker AhTE0357 with *AhMITE1* insertion polymorphism at 79 bp downstream of the gene *Araip*.*TG1BL* on chromosome B02, could differentiate the genotypes with sequential and alternate branching pattern to an extent of 32.8%. AhTE0357 was also associated with days to 50% flowering and number of primary and secondary branches. But selection for early maturing genotypes using AhTE0357 was not expected to bring any yield advantage since days to 50% flowering recorded negative and significant correlation with productivity and oil quality traits. AhTE0391, apart from being associated with the contents of oil, oleic acid, linoleic acid and palmitic acid, also showed association with protein content. The marker locus corresponding to AhTE0391 coincided with the gene *Aradu*.*7N61X*, which codes for alpha-glucosidase. In germinating grains of barley, *HvAGL97* codes for α-glucosidase, which is a major endosperm enzyme. It catalyzes conversion of maltose to glucose, but is not required for starch degradation [32, 33].

Test weight and pod width were positively and significantly correlated, and both showed strong association with AhTE0025, which corresponded to the gene *Aradu*.*7065G* on A07 chromosome coding for aldo/keto reductase family oxidoreductase. In *Digitalis purpurea*, aldo/keto reductase is known take part in the biosynthesis of cardiac glycosides [34]. In the previous studies, QTL regions for test weight were mapped at a distance of 1.5 Mb and 29.1 Mb by Fonceka et al. [35] and Huang et al. [36], respectively from the gene *Aradu*.*7065G* on A07 chromosome. Thus, *Aradu*.*7065G* on A07 chromosome could be a candidate gene for test weight.

The genomic resources (QTL regions/genes/markers) developed in this study help in generating the genetic resources to breed for improved peanut varieties. But the RILs used in this study directly provide an opportunity to select the superior lines for productivity traits. RILs 2-14(a)(ii), 2-29(g), 2-33(a) and 2-23(c) were superior since they recorded 7–15%, 35–50% and 1–4% increase over TMV 2 for pod yield, test weight and oil content, respectively. They also had 12–21% O/L higher oleate than TMV 2. They matured one to two days earlier than TMV 2. These RILs are being multiplied and evaluated in detail for variety development and commercial release.

The linkage map of TMV 2 × TMV 2-NLM, QTL regions and markers identified in this study for taxonomic, agronomic and productivity traits will be of immense use for fine-mapping and identifying the candidate genes. The loci of TE markers both at genic and non-genic regions are known to influence the gene expression [37], thereby favoring gene discovery for the taxonomic and productivity traits for use in peanut improvement. Further, the superior RILs identified in this study form the novel genotypes, which would be useful in peanut breeding for productivity and nutritional traits.

## Conclusions

QTL mapping using 432 recombinant inbred lines derived from TMV 2 and its mutant, TMV 2-NLM could identify the major QTL for days to 50% flowering, number of primary and secondary branches, test weight, pod width, protein and oil content, oleic acid, linoleic acid, palmitic acid, stearic acid, and behenic acid. Based on the three markers (AhTE0357, AhTE0391, AhTE0025) detecting *AhMITE1* insertion polymorphism, three genes *Araip*.*TG1BL* (B02 chromosome), *Aradu*.*7N61X* (A09 chromosome) and *Aradu*.*7065G* (A07 chromosome), respectively were identified for their association with taxonomic, productivity and quality traits.

## Supporting information

S1 Table List of AhTE markers with position and annotation. (XLSX)

S2 Table Features of the genetic map of TMV 2 × TMV 2-NLM population. (XLSX)

S3 Table Genetic map of TMV 2 × TMV 2-NLM. (XLSX)

S4 Table ANOVA for agronomic, disease resistance, productivity, nutritional and physiological traits in the RILs of TMV 2 × TMV 2-NLM. (XLSX)

S5 Table Mean, range and genetic variability components for agronomic, productivity, nutritional and disease resistance traits in RILs of TMV 2 × TMV 2-NLM. (XLSX)

S6 Table Correlation values for the disease resistance, productivity, agronomic, taxonomic, physiological and nutritional traits in the TMV 2 × TMV 2-NLM mapping population. (XLSX)

S7 Table Frequency distribution of RILs of TMV 2 × TMV 2-NLM for disease resistance, agronomic, physiological, productivity and nutritional traits. (XLSX)

S8 Table Single marker analysis for the disease resistance, productivity, agronomic, taxonomic, physiological and nutritional traits in rainy season-2014. (XLSX)

S9 Table Single marker analysis for the disease resistance, productivity, agronomic, taxonomic, physiological and nutritional traits in rainy season-2015. (XLSX)

S10 Table Quantitative trait loci analysis for the disease resistance, productivity, agronomic, taxonomic, physiological, nutritional traits in rainy season-2014. (XLSX)

S11 Table Quantitative trait loci analysis for the disease resistance, productivity, agronomic, taxonomic, physiological, nutritional traits in the mapping population in rainy season-2015. (XLSX)
